# A Case Report on an Atypical Presentation of the Syndrome of Irreversible Lithium-Effectuated Neurotoxicity (SILENT) in a War Veteran with Bipolar Disorder and PTSD

**DOI:** 10.1155/2020/5369297

**Published:** 2020-06-04

**Authors:** Miguela Marie Señga, Gemmalynn Sarapuddin, Edmundo Saniel

**Affiliations:** ^1^The Medical City, Ortigas Avenue, Pasig City, Philippines; ^2^Quirino Memorial Medical Center, Quezon City, Philippines; ^3^St Luke's Medical Center, Quezon City, Philippines

## Abstract

**Background:**

Lithium is still the first-line agent for bipolar disorder. Despite common knowledge on monitoring lithium levels to prevent toxicity, it still occurs at varying degrees. Here we present a rare sequela of lithium toxicity, the Syndrome of Irreversible Lithium-Effectuated Neurotoxicity (SILENT). *Case Presentation*. A 56-year-old male war veteran who is fully functional despite being on chronic lithium therapy for Posttraumatic Stress Disorder (PTSD) and bipolar disorder presented at the emergency room with altered mental status and seizures associated with elevated lithium levels and renal insufficiency. Antiepileptic drugs were given for seizure control, and intermittent hemodialysis was done to clear the lithium. Despite clearance of the offending agent, the patient remained to have a generalized slowing on repeated EEG with only eye opening and nonpurposeful limb movements regained even after more than 2 months of lithium cessation.

**Conclusion:**

SILENT has been coined after reports of persistent neurologic deficits were seen in patients who experienced lithium toxicity more than 2 months after cessation of lithium. Chronic lithium therapy predisposes to gradual accumulation of lithium in the brain. Demyelination is the typically reported feature of SILENT. It can also leave the patient in a persistent encephalopathic state. Chronic lithium toxicity from failure of monitoring puts patients on lithium therapy at risk.

## 1. Introduction

Lithium remains to be the first line in the treatment of bipolar disorder [1, 2]. Due to its narrow therapeutic index, lithium toxicity is a common problem. It has an incidence of 0.01 per patient-years in one study done in a cohort of 1340 patients exposed to lithium between 1997 and 2013 [3]. Neurotoxicity from lithium can be reversible or irreversible and can be potentially fatal [4]. Hence, monitoring of lithium levels cannot be overemphasized. The syndrome of irreversible lithium-effectuated neurotoxicity (SILENT) has been coined after case reports of persistent neurologic deficits after lithium toxicity despite normalization of lithium levels [5]. It is a rare sequela of lithium toxicity. We report a case of SILENT whose sequela is different from those in prior literature.

## 2. Case Presentation

**Figure 1:**
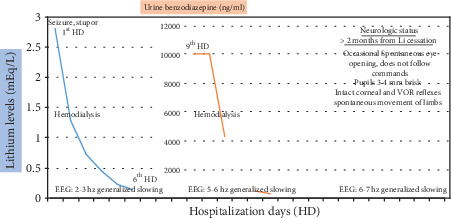
Timeline of the patient's neurologic status and serum lithium and urine benzodiazepine levels. HD: hospitalization day; Li: lithium; EEG: electroencephalogram; hz: Hertz.

A 56-year-old hypertensive and diabetic Filipino male who is a war veteran and has a known case of PTSD and bipolar disorder (type 1) was brought to the emergency room due to altered mental status. At baseline, the patient is fully functional until three weeks prior when he was reported to have episodes of agitation and night time awakening reliving his experience during the war. During this time, he was also noted to have increased consumption of fluids. He was reported to be wandering at night carrying his luggage believing that he will be taking a plane to America. He was brought to a psychiatric facility where his maintenance medications—risperidone, clonazepan, biperiden, and lithium carbonate—were continued. He continued to have agitation episodes, and his lithium dosage was increased from 600 mg to 900 mg per day. However, 2 days prior to admission, he had increasing sleeping time and with no oral intake. He was brought to our emergency room where he presented with generalized seizures and went on to status epilepticus. His vital signs were as follows: BP 120/60 mmHg, heart rate 137/min, respiratory rate 24/min, and temperature 36.7°C with no other abnormal systemic physical exam findings. Interictally, the patient was obtunded with a Glasgow coma score of 8 (E2V1M4). There were no apparent cranial nerve abnormalities, no focal weakness, and no nuchal rigidity noted. Initial work-up showed hypoglycemia (57 mg/dL), elevated lithium (2.8 mEq/L, therapeutic range 0.8-1.2 mEq/L), elevated creatinine (10.54 mg/dL), elevated potassium (5.4 mEq/L), and low sodium (130 mEq/L), and ABGs showed combined metabolic and respiratory acidosis. A plain cranial CT scan was done but was unremarkable. The patient was given intravenous valproic acid and midazolam drip for seizure control. The patient was deemed to have lithium toxicity. He underwent intermittent hemodialysis to clear the lithium. All psychiatric medications including lithium were discontinued. An electroencephalogram (EEG) was done the following day which showed a generalized slowing of the background activity with occasional spike waves in the frontal region. After 48 hours, the patient was seizure free, and midazolam was turned off. Lithium levels were monitored with a decreasing level down to 0.14 mEq/L after 5 days. Kidney function was also improving. Sodium was corrected with care to avoid rapid correction. However, the patient's mental status did not improve so urine benzodiazepine levels were obtained which was elevated at >10,000 ng/mL. Intermittent hemodialysis was continued and urine benzodiazepine level was monitored until it was below the threshold. Despite clearance of benzodiazepine and lithium (now at 0.01 mEq/L), the patient did not regain full consciousness. At best, the patient had eye opening and withdrawal to pain. Subsequent EEGs still showed a generalized theta slowing and with no more epileptiform discharges seen. Likewise, repeat cranial CT scan did not show any new findings. An MRI was not done due to a relative contraindication of the presence of a lodged bullet. He was voiding adequately no longer requiring hemodialysis. After 2 months of lithium cessation, the patient can now open his eyes and move his limbs spontaneously, but he still does not follow commands and has no purposeful activity. After more than 3 months, the patient still had the same mental status.  Figure 1 shows the trajectory of lithium levels against the neurologic status of the patient.

## 3. Discussion

The syndrome of irreversible lithium-effectuated neurotoxicity (SILENT) has been coined after case reports of persistent neurologic deficits after lithium toxicity despite normalization of lithium levels. Long-term neurologic sequelae are considered persistent when symptoms are present for at least 2 months after the cessation of lithium. In a review of 90 cases of patients with persistent deficits, the typical clinical profile identified are as follows: persistent cerebellar dysfunction, persistent extrapyramidal syndrome, persistent brainstem dysfunction, or dementia with varying organic mental syndromes. It also has atypical presentations which includes downbeat nystagmus, retrobulbar optic neuritis, persistent papilledema, choreoathetoid movements, peripheral neuropathy (both motor and sensory), myopathy, and blindness (due to central pontine myelinolysis). Only one case reported encephalopathic illness as a deficit (Table 1) [5].

Our patient initially presented with seizures and altered mental status which can be attributed to lithium toxicity, uremia, and hypoglycemia. However, with correction of all the metabolic problems, the patient's encephalopathy did not improve. This persistent encephalopathy may be another atypical manifestation of SILENT.

Lithium is widely distributed in most body tissues but with an uneven rate of distribution among different tissue compartments. It does not undergo liver metabolism but is renally cleared. Its half-life varies according to age and glomerular function. Lithium toxicity may occur with excessive intake or impaired excretion seen with renal insufficiency, low sodium diet, and congestive heart failure [3, 6, 7]. In a retrospective analysis of 97 cases of lithium poisoning over a 13-year period, neurotoxicity is concluded to be an iatrogenic illness, occurring in patients who have identifiable clinical risk factors: nephrogenic diabetes insipidus, older age, abnormal thyroid function, and impaired renal function [8]. Some of these were seen to be the same predisposing factors in our patient.

The distribution of lithium from the blood into the brain is slower relative to its distribution into the kidneys, muscle, and bones. In a study done on adult nonpregnant albino rats given 5 mEq/kg body weight of lithium, the most rapid rise in lithium concentration was seen in kidney tissue while the maximum lithium concentration in the brain was only reached until about 24 hours after its administration. This is presumed to be due to its slow passage across the blood brain barrier [9]. In a similar fashion, lithium is slowest to clear from the brain.

Lithium toxicity may be differentiated in terms of the temporality of exposure to lithium. Acute is seen in those who overdose on lithium as in suicide, chronic is when a patient on maintenance lithium suffers a reduction in kidney function, and acute-on-chronic is when a patient maintained on lithium suddenly ingests a large amount all at once [6, 10]. Long-term lithium use is associated with an increased risk of loss of renal function which further increases serum lithium levels [3, 7]. Our patient presented with decreased renal function presumably from chronic lithium exposure and his long standing hypertension and diabetes. This further decreases the clearance of lithium. Lithium toxicity manifests systemically with gastrointestinal, cardiac, and renal symptoms.

Most frequently, acute toxicity initially results in GI symptoms such as nausea, vomiting, and diarrhea [6]. Patients on chronic lithium therapy develop toxicity gradually and often present with neurologic findings.

Symptoms of toxicity may be classified with varying serum lithium levels, mild (1.5-2.5 mEq/L) and moderate (2.5-3.5 mEq/L), and with severe central nervous manifestations when levels exceed 3.5 mEq/L. Hansen and Amisden classified the severity of toxicity according to its neurological presentation. Mild toxicity presents with nausea, vomiting, lethargy, tremor, and fatigue, while moderate toxicity may present with confusion, agitation, delirium, tachycardia, and hypotonia, and severe toxicity presents with coma, seizures, hyperthermia, and hypotension [6, 10].

As time is required for lithium to penetrate the CNS, neurologic effects from lithium develops late in acute lithium poisoning but is more often present with chronic lithium toxicity. The duration of lithium exposure correlates with the incidence and severity of lithium neurotoxicity. Severe lithium neurotoxicity occurs almost exclusively in the context of chronic therapeutic administration of lithium [3]. In the background of chronic lithium exposure, the rise in lithium levels has started the cascade of events leading our patient to this encephalopathic state. Demyelination caused by lithium at multiple sites in the nervous system is hypothesized to be the cause of SILENT [5].

There is no unified consensus on the lithium level that requires initiation of hemodialysis in lithium-intoxicated patient, and no clinical trials were done. Some studies recommend initiating hemodialysis at 2.5 mEq/L when a patient shows severe signs of lithium toxicity or when there is an ongoing renal impairment that prevents is excretion [11].

Our patient presents with symptoms of severe lithium toxicity despite serum lithium level at 2.8 mEq/L. It persisted even long after levels normalized with hemodialysis. The absolute serum lithium level or quantity of lithium ingested does not correlate with the severity of toxicity [12].

## 4. Conclusion

SILENT is a disabling but preventable neurologic sequela of lithium toxicity. It may leave a patient in a persistent encephalopathic state. Close monitoring of lithium levels in those on chronic lithium therapy and identification of risk factors for toxicity cannot be overemphasized. Despite common knowledge of lithium toxicity, rare cases like this still occur for which clinicians should look out for. Families who care for patients taking lithium must be educated on vigilant follow-up and recognition of signs of toxicity.

## Figures and Tables

**Table 1:** Patients with persistent encephalopathic illness as a sequela of lithium toxicity.

Author	Age/gender	Persistent sequelae	Precipitating factors	Dose, mg/day	Plasma level mM/L	Acute neurologic signs
Lecamwasam et al. [13]	71/M	Encephalopathic illness with histologic evidence of neurologic sequelae	Chronic lithium toxicity	1500 mg	0.88-0.99	Parkinsonism, dysphagia, dysarthria, deterioration in mobility, coma, generalized tremor
Our patient	56/M	Prolonged encephalopathy Persistent slowing of the background activity of EEG	Chronic lithium therapy	900 mg	2.8	Seizure, altered mental status

## Abbreviations

SILENT: Syndrome of Irreversible Lithium-Effectuated Neurotoxicity
PTSD: Posttraumatic stress disorder
EEG: Electroencephalogram
CT: Computed tomography.
